# The Role of EFL Teachers' Self-Disclosure as Predictors of Student's Willingness to Communicate and Their Engagement

**DOI:** 10.3389/fpsyg.2021.748744

**Published:** 2021-09-17

**Authors:** Xingpei Liu, Linhan Zhu

**Affiliations:** ^1^School of College English Teaching and Research, Henan University, Kaifeng, China; ^2^School of Journalism and Communication, Lanzhou University, Lanzhou, China

**Keywords:** self-disclosure, willingness to communicate, engagement, second language (L2), pedagogical implications

## Abstract

This study aims to delve into the role of teachers' self-disclosure on developing students' willingness to communicate (WTC) and students' engagement. First of all, a definition of willingness to communicate is proposed, then the concept of teachers' disclosure is explained. Moreover, the definition of engagement and its facilitators are mentioned. The interpersonal relationship between students and teachers with regard to some examples is discussed then. Finally, the significant effect of what has been disclosed by the teachers in both students' willingness to communicate and engagement is discussed. Some limitations in this line of study and pedagogical implications are proposed for avid researchers.

## Introduction

Emotions play a pivotal role in language learning (Dewaele, 2015). When we look at the second language through the lens of willingness to communicate, many concepts can be highlighted, for instance, teachers' self-disclosure (MacIntyre et al., 1998). Languages are learned by people whose motivations, emotions, and relationships are integrated into every step of the learning and communicating process. The interpersonal relationship in which both teachers and students are involved is what has attracted attention in recent studies since students' engagement and academic outcomes in all activities and all aspects throughout the class are highly impacted by teachers' behavior. Seven examples of positive teacher interpersonal communication behaviors such as “teacher care, clarity, credibility, rapport with students, stroke, immediacy, and confirmation” and its impact on students' academic outcomes, for instance, the amount of motivation, the amount of engagement, learning process, their willingness to communicate, their accomplishments, and the amount of success (Xie and Derakhshan, 2021). This study is a review of the role of teachers' self-closure on both students' willingness to communicate and students' engagement. First and foremost, the term “willingness to communicate” has been defined. Then self-disclosure has been discussed from different aspects. Then, the interpersonal relationship between students and teachers which is of paramount importance has been dealt with. Last but not least, the crucial role of teachers' disclosing information on students' participation in activities and willingness to communicate has been analyzed.

## Background

### Willingness to Communicate

**Figure 1:**
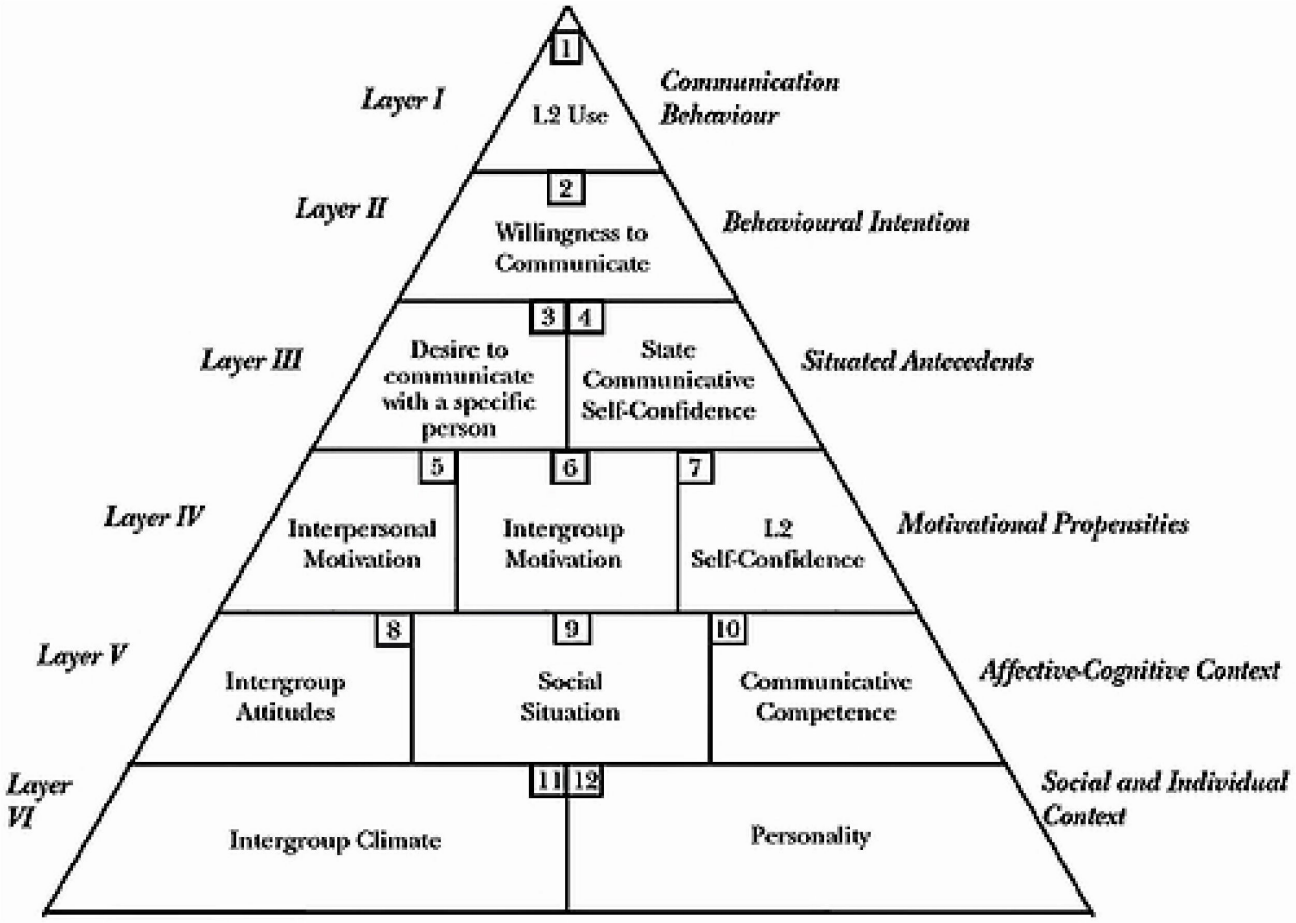
The heuristic model of variables influencing WTC (MacIntyre et al., 1998, p. 547).

The concept of willingness to communicate and its relevance to the first language was first introduced by McCroskey and Baer (1985), and its nature has been said to be situation-dependent. Then L2 WTC was defined by MacIntyre et al. (1998) as “readiness to enter into discourse at a particular time with a specific person or persons, using a L2” (p. 547). It was claimed that willingness to communicate will be enhanced if the learners should risk giving up their safety zone, for instance, they may have difficulty starting a conversation with others, using L2 (Zarrinabadi and Pawlak, 2021). A model, Figure 1, was put forward by MacIntyre et al. (1998). There are 6 layers in this pyramid that affect the willingness to communicate. The top three layers are said to be context-specific and have a temporary effect on WTC, while the lower three layers are believed to be very practical and have a long-lasting impact on WTC. Layer 6 indicates that WTC is affected by learners' characteristics and the relationships between language groups and there can be seen a slow change in both factors (MacIntyre, 2020). Personality traits such as extraversion impact on WTC. The more extroverted people are, the more willing they are to communicate (Fatima et al., 2020). Layers 4 and 5 show that WTC is also affected by interpersonal, intrapersonal, social, and contextual factors. As a brief example, having optimistic attitudes toward both the language and people speaking it are found to heightened WTC (Ghonsooly et al., 2012). Or about the classroom contexts, the more positive and supportive the relationship between the teachers and students are, the higher WTC would be (Cao, 2011). Layer 3 of this model comprises the passion to communicate with a specific person and confidence which is raised due to putting in such a situation. In layer 2, there is only one component willingness to communicate. As a result, there might be fluctuations in using L2 (MacIntyre, 2007). Students are reluctant to speak a second language unless they are willing to communicate.

### Teachers' Self-Disclosure

This term has been defined by Cosby (1991) as information that one discloses or reveals about himself. Self-disclosure aids people in building new bounds and keeping the ones that they have already had (Collins and Miller, 1994). Learners and teachers sometimes discuss their personal problems in the classroom. Some personal stories and experiences are shared by the teachers in their classes while teaching the subject-matter (Nussbaum et al., 1987). This type of communication is known as teacher self-disclosure, described as “conscious and deliberate disclosures about one's self, aspects of one's professional practice, world or personal views, personal history, and responses to ongoing classroom events” (Rasmussen and Mishna, 2008). It has also been proposed by Lannutti and Strauman (2006) that self-disclosure varies from context to context, for example, the communication that is shared by teachers through the class is really different from the one which is pleasurable in personal relationships since the latter one needs to be illustrative rather than revealing. Furthermore, in education, a teacher's self-disclosure is perceived as the sentences that a teacher says about himself which may not be pertinent to the subject that is the focus of the class (Sorensen, 1989). Teachers' self-disclosure is related to a number of positive learning results (Cayanus and Martin, 2016; Henry and Thorsen, 2021). Teachers who talk about their behaviors, share some stories about their personal lives and experiences and also their personal beliefs and values, have been found to increase the students' perception of subject knowledge (Wambach and Brothen, 1997), and students' passion about the learning (Sorensen, 1989). In addition, Teachers' self-disclosures are associated with students' motivation and engagement (Cayanus and Martin, 2016). Students are more inclined to become engaged in the activities when teachers share their own stories (Zhang et al., 2008), it also maximizes the levels of engagement, and boosts students' interest in the subjects they are going to learn, the students' motives for communication additionally enhances (Cayanus and Martin, 2008).

### Engagement Facilitator

How actively learners participate in the activities is called engagement. It is highly unlikely to attain meaningful learning without being engaged. Engagement has been said to be context-based, for example, learners' culture, family, school, peers affect their engagement (Hiver et al., 2021). Six items are found to facilitate the engagement. The first facilitator is authenticity, for authenticity to be met in learning contexts, teachers should know their students' needs and interests to choose the tasks that meet the students' requirements. The second facilitator of engagement is social interaction. The word “social” here refers to the interaction between a well-educated interlocutor who can provide the learners with productive, creative feedback, and the students. Additionally, here the focus is shifted to both competitions and collaboration depending on what works in that specific learning context. The third facilitator is learning support. The students are highly likely to be engaged in the tasks when they know that they are supported. This support is mostly teacher-based even though it can be given by peers and experts. Support can be seen in the form of obvious, attainable goals, available resources, enough time, and support that are supposed to be shared. Another facilitator is students' interest. In terms of students' interests, what can be stressed is individual differences. Some students are interested in writing a paragraph, while others are keen on making posters to learn new items. Autonomy is said to be another facilitator which means the learner has control over the learning aspects that he needs. Last but not least, the level of difficulty of a task or challenge in a task is seen as another facilitator for language learners. If students face the insufficient challenge, they may feel absolutely tired. As opposed to what has been said, if they encounter too much challenge, it maximizes a lack of confidence or a sense of frustration. All the six factors facilitating the engagement in the classroom are among the interpersonal factors (Egbert, 2020). Needless to say, engagement is an indispensable part of the process of learning and a multifold phenomenon. It has been categorized into different classifications: Behavioral engagement such as the amount of effort and the voluntary involvement in speaking; emotional engagement such as positive feelings and autonomous approach to learning; cognitive engagement such as non-verbal cues like body language, facial expression, and eye contact; social engagement such as collaborative activities with others (Hiver et al., 2021).

### Teacher-Student Interrelationship

According to Wang et al. (2021), the impact of students' optimism on their development is considered important and it needs to be taken into consideration. Moreover, seven examples of positive psychology variables have been mentioned which go as follows: “academic engagement, emotion regulation, enjoyment, grit, loving pedagogy, resilience, and well-being.” These are said to widely impact on experiencing productive L2 learning.

The interpersonal relationship between the teachers and students have been gaining much attention due to the fact that it helps learners to cope with their stress and take risks in the learning process such as starting to communicate in the second language, as well as developing socially and emotionally through practicing a new language, and also acts as a companionship in shared tasks (Martin et al., 2009; Xie and Derakhshan, 2021). Teachers play a paramount role in students' academic and non-academic development. It has been revealed by the students that when teachers care for their students, the students can learn more since they feel valued. When students feel that they are accepted by their teachers, their positive cognitive, emotional, and behavioral engagement are dramatically enhanced (Connell and Wellborn, 1991). Teachers encourage students to be autonomous and develop a sense of motivation in their students as well. A healthy emotional, social, and intellectual function, in addition to the positive sense of self-worth and self-esteem are fostered by positive interpersonal relationships between both teachers and students (Martin and Dowson, 2009).

### The Effect of Teachers' Self-Disclosure in Willingness to Communicate and Students' Participation

It was pinpointed that both teachers' self-disclosure and students' social desire to their teachers are positively correlated. When learners see the self-closure in their teacher, they feel more motivated to make progress and find the class atmosphere more productive (Cayanus and Martin, 2008). According to Goldstein and Benassi (1994), the more the amount of teacher self-disclosure, the more the amount of students' classroom engagement would be. Therefore, the conclusion that can be drawn out of the afore-mentioned points is that a teacher disclosing his ideas, builds trust, and thus, entice the students to participate in classroom discussion. This result is in line with the consequences that have been found by Cayanus et al. (2009) recommending that teacher self-disclosure impacted the relationship between the teachers and students positively, and also communication through the class, and makes learners able to participate more in the classroom.

## Implications and Future Directions

Both interpersonal and intrapersonal factors are of central importance in the context of learning and teaching. In the present study, the role of teachers' self-disclosure -TDS- in both students' willingness to communicate and students' engagement has been examined. Although many studies have been conducted to consider the significant role of TDS, little attention was laid on its details and impacts from different viewpoints. Teachers' self-disclosure deserves more attention from different aspects, leading to better academic outcomes that can be the focus of other future studies. For instance, the effect of teacher self-disclosure in students' bravery to make mistakes in the learning context would be a new research line for avaricious people. As it was studied, making mistakes is an inseparable part of learning a new language and without which the ability to speak a new language cannot be strengthened (Gregersen and Horwitz, 2002). It inevitably happens when learning a new language and those mistakes had better be embraced and welcomed with open arms so as to allow learners to enjoy the complete process of learning. As to my own teaching context, I have been teaching those who major in Soprts Education, Fine Arts and Musicology in my university, whose English proficiency cannot be compared with those who study other majors. However, I have attempted to build up peace and harmonious interpersonal relations with them, so they are more willing to express their ideas in the classrooms. In other words, with regard to self-disclosure, the more teachers' experiences about their own process of learning a new language is shared, the more willing the students may become to accept their mistakes, which is a big part of learning. As a consequence, a qualitative in-depth study should be done to find the correlation between these two important factors.

Form a pedagogical point of view, teachers' awareness should be raised to enhance students' interests in the process of learning and being engaged in the activities, leading to a better educational system that enables the students to enjoy the learning process more and causes the teachers to be fully aware of students' needs, wants, and interests. So far, little attention has been focused to conduct studies, considering qualitative research in which educating teachers is the first priority and its effects on students' academic outcomes will be identified. In doing so, a longitudinal study could be carried out which even deepens the accuracy of the study to scrutinize the above-mentioned points.

## Author Contributions

XL drafted the first manuscript and LZ revised the layout and analyzed the figure. Both authors contributed to the article and approved the submitted version.

## Conflict of Interest

The authors declare that the research was conducted in the absence of any commercial or financial relationships that could be construed as a potential conflict of interest.

## Publisher's Note

All claims expressed in this article are solely those of the authors and do not necessarily represent those of their affiliated organizations, or those of the publisher, the editors and the reviewers. Any product that may be evaluated in this article, or claim that may be made by its manufacturer, is not guaranteed or endorsed by the publisher.
